# Drug-Repurposing Screen Identifies Thiostrepton as a Novel Regulator of the Tumor Suppressor DAB2IP

**DOI:** 10.3390/biom15081147

**Published:** 2025-08-08

**Authors:** Rossella De Florian Fania, Serena Maiocchi, Raffaella Klima, Monica Rossin, Valeria Pellegrini, Sabrina Ghetti, Davide Selvestrel, Maria Chiara Mattevi, Luca L. Fava, Luca Braga, Licio Collavin

**Affiliations:** 1 Department of Life Sciences, University of Trieste, Via L. Giorgieri 1, 34127 Trieste, Italy; 2 International Centre for Genetic Engineering and Biotechnology (ICGEB), Padriciano 99, 34149 Trieste, Italy; 3 Armenise-Harvard Laboratory of Cell Division, Department of Cellular, Computational and Integrative Biology (CIBIO), University of Trento, Via Sommarive 9, 38123 Povo, Italy; 4 Unit of Molecular Oncology, Centro di Riferimento Oncologico di Aviano (CRO) IRCCS, National Cancer Institute, Via Gallini 2, 33081 Aviano, Italy

**Keywords:** high-throughput screening, AIP1, prostate cancer, tumor suppressor genes, Ras-GAP, FOXM1, HiBiT, protein tagging, CRISPR-Cas9

## Abstract

The tumor suppressor DAB2IP, a RasGAP and cytoplasmic adaptor protein, modulates signal transduction in response to several extracellular stimuli, negatively regulating multiple oncogenic pathways. Accordingly, the loss of DAB2IP in tumor cells fosters metastasis and enhances chemo- and radioresistance. DAB2IP is rarely mutated in cancer but is frequently downregulated or inactivated by multiple mechanisms. Solid experimental evidence shows that DAB2IP reactivation reduces cancer aggressiveness in tumors driven by multiple different oncogenic mutations, making this protein an interesting target for cancer therapy. Considering this evidence, we screened a drug library to identify molecules that increase DAB2IP protein levels. We employed CRISPR/Cas9 gene editing to generate two prostate cancer cell models in which endogenous DAB2IP is fused to HiBiT, a peptide tag that enables luminescence-based detection of protein levels in a sensitive and quantitative manner. Using this approach, we identified drugs able to increase DAB2IP levels. We focused our attention on thiostrepton, a natural cyclic oligopeptide antibiotic that has been reported to inhibit the survival of various cancer cell lines. Functional experiments revealed that the cancer-inhibitory effect of thiostrepton is reduced in the absence of DAB2IP, suggesting that upregulation of this protein contributes to its action. These findings encourage further development of thiostrepton for the treatment of solid cancers and unveil a novel molecular target underlying its anti-tumoral activity.

## 1. Introduction

Prostate cancer is one of the most common cancers in men; with an age-related incidence, it is the second leading cause of cancer death in men in the United States [1]. Although current therapies are often effective, there are patients who do not completely recover from the disease and develop resistance to chemo- and radiotherapy, rendering the treatment inefficient or even detrimental. Novel strategies to counteract the intrinsic causes of such resistance could significantly improve future therapies.

DAB2IP (Disabled-2 Interacting Protein) is a cytoplasmic Ras GTPase-activating protein (GAP) and adaptor involved in signal transduction by multiple inflammatory cytokines and growth factors, negatively modulating key oncogenic pathways such as TNF/NF-kB, WNT/β-catenin, PI3K/AKT, and androgen receptors [2,3,4]. DAB2IP is also known as ASK1-interacting protein (AIP1), given its role in inducing the release of ASK1 from the inhibitory binding of 14-3-3 in response to TNF-α, favoring the subsequent activation of the pro-apoptotic ASK1-JNK pathway [5].

The loss of DAB2IP function represents an advantage for cancer initiation and progression, and accordingly, it is frequently downregulated in various human malignancies [4,6,7]. Intriguingly, DAB2IP is rarely inactivated by deletion or mutation but is preferentially inhibited at the transcriptional and post-transcriptional level via different mechanisms [4].

Various studies demonstrated that restoring DAB2IP expression in DAB2IP-depleted cancer cells reverses metastatic behavior and drug resistance both in vitro and in vivo, in multiple tumor types. Based on this evidence, DAB2IP can be considered a strong candidate for the development of therapeutics aimed at increasing its protein levels in cancer cells, thus restoring its onco-suppressive role. In fact, even a moderate increase in DAB2IP levels may be able to limit cancer aggressiveness, as we previously demonstrated by targeting some known mechanisms of DAB2IP inactivation [8,9,10].

Building upon the aforementioned concepts, in the present study, we tested 1280 drugs, the majority of which are already approved by the Food and Drug Administration (FDA) or the European Medicines Agency (EMA), to evaluate their potential to increase DAB2IP levels in prostate cancer cell lines. To monitor the amount and dynamics of the endogenous DAB2IP protein through a sensitive, quantitative, and high-throughput assay, we labeled endogenous DAB2IP with the luminescence-based tag HiBiT [11,12]. Using this approach, we uncovered candidate molecules able to modulate DAB2IP levels in cancer cells. We focused on thiostrepton, finding evidence that DAB2IP upregulation can contribute to its ability to counteract the aggressiveness of cancer cells.

## 2. Materials and Methods

### 2.1. Cell Lines, Transfections, and Drug Treatments

PC3 (human prostate adenocarcinoma; RRID:CVCL_0035), DU145 (human prostate carcinoma; RRID:CVCL_0105), and MCF7 (human breast adenocarcinoma; RRID:CVCL_0031) cells were purchased from ATCC.

PC3 were cultured in DMEM-F12 (1:1) (ECM0728L, ECM0135L, Euroclone, Milano, Italy) supplemented with 10% FBS, pen/strep solution, MEM Nonessential Amino acids (ECB3054D, Euroclone, Milano, Italy), and Sodium Pyruvate (1 mM), (ECM9542D, Euroclone, Milano, Italy). DU145 were cultured in EMEM (LOBE12611F, Euroclone, Milano, Italy), supplemented with 10% FBS and pen/strep solution. MCF7 were cultured in EMEM (LOBE12611F, Euroclone, Milano, Italy), supplemented with 10% FBS, pen/strep solution, and human insulin 10 ug/mL (I2643, Merck Life Science, Milano, Italy). H1299 cells were cultured in RPMI (ECM2001L, Euroclone, Milano, Italy) supplemented with 10% FBS, and pen/strep solution. Cells were cultured at 37 °C in a humidified incubator with 5% CO_2_. All cell lines were periodically tested for the absence of mycoplasma contamination.

For knockdown experiments, cells were transfected the same day of plating (384-well plates) or 24 h after plating (6-well plates) with 50 nM siRNA (384-well plates) or 40 nM siRNA (6-well plates) or 3 nM miRNAs (siRNA sequences in Table 1). Cells were processed after 48 h, except when otherwise specified. DAB2IP silencing was performed using a mix of siDAB2IP A and siDAB2IP B. FOXM1 silencing was performed using a specific siRNA (siFOXM1 #1) or a commercial pool of three different siRNAs (siFOXM1 #2).

For validation experiments, cells were treated with ubenimex (CAS-58970-76-6), thiostrepton (CAS-1393-48-2), and salmeterol (CAS-89365-50-4) from the Prestwick Chemical library (Greenpharma, Orléans, France). Additional experiments and all phenotypic assays were performed using thiostrepton from Calbiochem (Merck Life Science, Milano, Italy). In all experiments, cells were treated with drugs or with an equivalent volume of DMSO for 36 h, unless otherwise indicated.

### 2.2. High-Throughput Screening

The screening was performed using a clone of PC3-HiBiT cells with homozygous insertion of the tag. Cells (4.0 × 10^3^ per well) were seeded in white opaque 384-well microplates [6007690, Revvity, Milano, Italy], 24 h later 1280 compounds [Prestwick Chemical Library^®^, Greenpharma, Orléans, France] were transferred robotically from library stock plates to the plates containing the cells at the final concentration of 10 μM; as a control, 1% DMSO was added to columns 1, 2, 23 and 24 of each plate. Cells were processed 36 h after the addition of drugs. Briefly, wells were aspirated with a Plate washer BioTek 405, leaving 10 μL of medium. A total of 10 μL of 2X CellTiterFluor reagent (Promega Italia, Milano, Italy) was added to the cells and incubated for 30 min at 37 °C, 5% CO_2_. Fluorescence was detected using EnVision multimode plate reader with dual monochromator [Revvity, Milano, Italy] (400 nm_Ex_/505 nm_Em_). Immediately after, 20 μL of 2X NanoGlo Lytic reagent (Promega Italia, Milano, Italy) was added to the wells. The cells were incubated for 10 min at room temperature, and luminescence was detected with an EnVision multimode plate reader. Luminescence values were normalized on fluorescence readings. The screening of all 1280 compounds was performed once, then the top hits were selected and thoroughly analyzed separately.

### 2.3. Genetic Modification by CRISPR/CAS9

To generate knock-in cell lines (HiBiT KI), a sgRNA was designed for insertion of a donor sequence at the C-terminus of endogenous DAB2IP, immediately before the last splice site. A single-stranded donor DNA (ssDNA) was synthesized with left and right homology arms and a central region encoding the HiBiT peptide, preceded by a short flexible linker and followed by a stop codon (Figure S1C). Ribo-nucleo-protein (RNP) complexes with sgRNA and recombinant Cas9 were prepared and assembled with donor ssDNA exactly as described in [13]. PC3 and DU145 cells were electroporated in 16-well Nucleocuvette Strips (Lonza) using a Lonza 4D-Nucleofector (AAF-1002B, Euroclone, Milano, Italy). More details are provided as Supplementary Materials.

To generate knock-out cell lines (DAB2IP KO1 and KO2), two different sgRNAs were designed targeting exon 6, common to all predicted DAB2IP isoforms. RNP complexes with recombinant Cas9 were prepared as above [13]. PC3 cells were nucleofected using a NEPA21 electroporator (NepaGene); 6.0 × 10^5^ cells per reaction were dispensed in electroporation cuvettes (NEPA EC-002S, 2 mm gap). Poring pulse was 175 V for a 2.5 ms pulse length twice with a 50 ms interval between the pulses and 10% decay rate with + polarity. Transfer pulse was five pulses at 20 V for 50 ms, with 50 ms interval between pulses and 40% decay rate with +/− polarity. The efficiency of gene editing was evaluated by analyzing Sanger sequencing results of PCR products of the target genomic region using TIDE (https://tide.nki.nl). Sequences of crRNAs and ssDNA are listed in Table 2, PCR primers for genomic DNA amplification are listed in Table 4.

### 2.4. Protein Expression Analysis

Cells were lysed in 2X SDS Sample buffer (125 mM Tris-HCl at pH 6.9, 4% SDS, 20% Glycerol, 3% β-mercaptoetanol). Samples were sonicated and incubated at 95 °C for 5 min. About 10–20 ug of proteins were separated on SDS-PAGE [BioRad Mini-Protean^®^ Tetra System] and transferred to nitrocellulose membranes [GE Healthcare, 10600001]. Membranes were blocked for at least 1 h at room temperature in 5% skim milk PBST (0.1% Tween-20 in PBS) and incubated overnight at 4 °C with primary antibodies diluted in 5% skim milk PBST (Table 4). Membranes were washed three times in PBST for 10 min. Then were incubated for 1 h at 4 °C with horseradish peroxidase (HRP)-conjugated secondary antibody diluted 1:4000 in 5% skim milk PBST. Membranes were washed as above and rinsed in PBS before detection. Protein detection was performed by chemiluminescence using Liteablot Extend [EMP013001, Euroclone] for DAB2IP and c-Myc, and with ECL [32209; Thermo Scientific] for normalization markers. A chemiluminescent signal was impressed on autoradiography Hyperfilm ECL [Amersham]. Densitometric band quantification was performed using ImageJ 2.16.0 software. Primary antibodies used are listed in Table 3.

### 2.5. RNA Expression Analysis

Total RNA was extracted with TRIFAST II [EMR517100, Euroclone] using a standard protocol. For RT-qPCR, 500 ng of total RNA was reverse transcribed with iScript Advanced cDNA Synthesis Kit (mRNA) [1725038, -Bio-Rad Laboratories, Milano, Italy]. Real-time PCR was performed using iTaq Universal SYBR Green SMX 5000 [1725124, Biorad] on a CFX96 Real-Time PCR System [Biorad]. Primers are listed in Table 4.

### 2.6. Colony Formation Assay

Cells were seeded at a density of 5000 cells per 6 cm diameter plate and incubated for 48 h in complete culture medium, then the cells were treated with thiostrepton at EC_50_ concentration for 48 h. After 10–14 days, the cells were fixed and stained with 10% methanol and 0.1% crystal violet [C-6158, Merck Life Science, Milano, Italy] in H_2_O for 30 min. Plates were photographed, and colony formation efficiency was calculated by measuring the % of colonies-covered area over the whole dish area. The areas were measured using ImageJ software.

### 2.7. Wound Healing Assay

Cells were seeded in 96-well plates and cultured to 90% confluence in the presence of Hoechst 33342 (1:50,000 dilution) [Thermo Fisher H3570, Life Technologies Italia, Segrate, Italy]. Cells were scraped with a sterile pipette tip, washed with PBS to remove detached cells, and treated with thiostrepton (or DMSO) at the EC_50_ concentration. The scratch area was photographed at defined time points, and cell migration was quantified and expressed as the average rate of closure of the scratch. Images of live cells were automatically acquired with PerkinElmer Operetta every 12 h up to 48 h. The width of the scratches was measured using ImageJ. Migration distances, M (t), were calculated as follows: M (t) = width (0)–width (t), where width (t) is the wound width at time t and width (0) is its initial width.

### 2.8. Matrigel Drop Invasion Assay

PC3 cells at 70% of confluence were pre-treated with thiostrepton at the EC_50_ concentration (3.3 uM), or with the corresponding volume of DMSO, for 48 h. Then, cells were trypsinized and 7500 cells per condition were plated in 12 μL drops of Matrigel (7 mg/mL) [BD Bioscience, SIAL, Roma, Italy] in low serum (0.1% FBS). Five drops of Matrigel with cells were plated for each condition and then coated with 2 mL of high serum (10% FBS) medium. After 7 days, the drops were fixed and stained with 10% methanol and 0.1% crystal violet in H_2_0. Evasion was measured using ImageJ 2.16.0 software on 5 random non-overlapping microscope fields at 100X magnification of four different drops for each condition.

### 2.9. Spheroid Assay

A total of 500 cells/well were seeded in round-bottomed ultra-low attachment 96-well plates in complete medium plus 2,5% Matrigel as previously described [14]. After 48 h, spheroids were treated with 3 μM or 10 μM of thiostrepton or DMSO as a control. Images of live spheroids were taken at the time of treatment (t0) and after 7 days (t) using ZEN Lite Software (version 3.2, Carl Zeiss Microscope GmbH, Jena, Germany) at 100× magnification. The area of spheroids was measured in arbitrary units using ImageJ software on 4–6 spheroids per condition. Spheroids’ growth was calculated as follows: Area (t)/Area (t0).

### 2.10. Analysis of Connectivity Map Datasets

Gene-expression data for PC3 and MCF7 treated with the various drugs, reported as weighted Z-scores, were downloaded from CLUE (https://clue.io). We then used the expression vector, calculated by computing the Spearman correlation among replicates as reported in the CLUE guidelines, to identify differentially expressed genes (DEGs). We considered all the genes with an adjusted *p*-value lower than 0.05 as significant. To compare the transcriptional impact of the different drugs, we sorted DEGs based on the absolute value of log fold change and analyzed the top 1500. For gene-set enrichment analysis, we used the ShinyGO platform (version 0.80, http://bioinformatics.sdstate.edu/go/, accessed on 29 May 2024).

### 2.11. Statistical Analysis

Normal distribution of data was assessed using the Shapiro–Wilk test or D’Agostino-Pearson test according to the sample size. Data with normal distribution were analyzed using two-tailed Student’s *t* test (for two groups) or with one-way or two-way ANOVA followed by Tukey’s or Dunnett’s or Sidak post hoc test (for more than two groups), as indicated in figure legends. Data with non-normal distribution were analyzed using the nonparametric Mann–Whitney test (for two groups) or Kruskal–Wallis test followed by Dunn’s multiple comparisons (for more than two groups), as indicated in figure legends. The number (*n*) of independent experiments is indicated in the figures or figure legends. Data were analyzed using Prism 7.0 (GraphPad). Statistical significance was defined as *p* < 0.05. Statistical significance is indicated using the following annotation: ns = non-significant (*p* ≥ 0.05), * *p* < 0.05, ** *p* < 0.01, *** *p* < 0.001, and **** *p* < 0.0001.

## 3. Results

### 3.1. Endogenous DAB2IP Tagging with HiBiT

To discover drugs that modulate DAB2IP, we tagged endogenous DAB2IP with the HiBiT peptide for quantitative, luminescence-based detection of protein levels under physiological conditions. HiBiT is an 11 amino acid peptide that can be complemented with a larger subunit, LgBiT, to reconstitute a functional NanoLuc enzyme [11,12]. Since strong evidence indicates that DAB2IP reactivation in prostate cancer counteracts metastasis and chemoresistance [15,16], we chose to edit PC3 and DU145 human prostate cancer cell lines; the two lines differ in DAB2IP expression levels, p53 status, and overall aggressiveness, rendering them suitable for exploring various mechanisms of DAB2IP regulation in cancer.

The human DAB2IP gene is complex. It contains multiple transcriptional start sites that encode at least three N-terminal variants of the protein; moreover, alternative splicing generates two possible C-termini (Figure S1A). Aiming for a C-terminal fusion of the tag, we analyzed the alternatively spliced variants and confirmed that both are expressed in several normal and transformed cell lines (Figure S1B); therefore, to tag all possible DAB2IP isoforms, we placed the HiBiT peptide followed by a stop codon immediately upstream of the last donor splice site, so that the same C-terminally tagged DAB2IP protein is translated regardless of alternative splicing of the last intron (Figure S1C).

To verify that the HiBiT peptide in this position was functional, we cloned the corresponding construct into a mammalian expression vector and confirmed that DAB2IP could be readily detected by luminescence in transfected cell lysates (Figure S1C,D).

We tagged endogenous DAB2IP with the HiBiT peptide using a CRISPR-Cas9 protocol previously described [13]. Bulk nucleofected cells were screened first by luciferase assay (Figure S2B) and then by PCR on genomic DNA (Figure S2C), confirming the successful integration of the HiBiT tag into the target region. PC3 and DU145 cells positive for HiBiT insertion were selected as single clones with homozygous DAB2IP tagging. Responsiveness of the model was evaluated by siRNA-mediated silencing of DAB2IP, and by transfection of DAB2IP-targeting miR-149-3p [8]. The reduction in luminescence was in line with that observed by Western blot, confirming that the system is applicable to measure variations in endogenous DAB2IP protein levels (Figure S2D).

### 3.2. High-Throughput Luminescence and Fluorescence-Based Screen Identifies Molecules Able to Modulate DAB2IP Expression Levels

To screen for molecules that increase DAB2IP protein levels, we used the more aggressive PC3 cells, which display lower basal expression of DAB2IP compared to DU145. To normalize luciferase activity for variability in cell numbers, due to uneven cell seeding or nonspecific toxicity, we quantified viable cells using a fluorescence-based assay (CellTiter-Fluor, Promega) that is compatible with subsequent luciferase quantification. We thus expressed DAB2IP levels as the ratio of Luminescence over Fluorescence intensity (Lum/Fluo) (Figure S2E–G).

**Figure 1:**
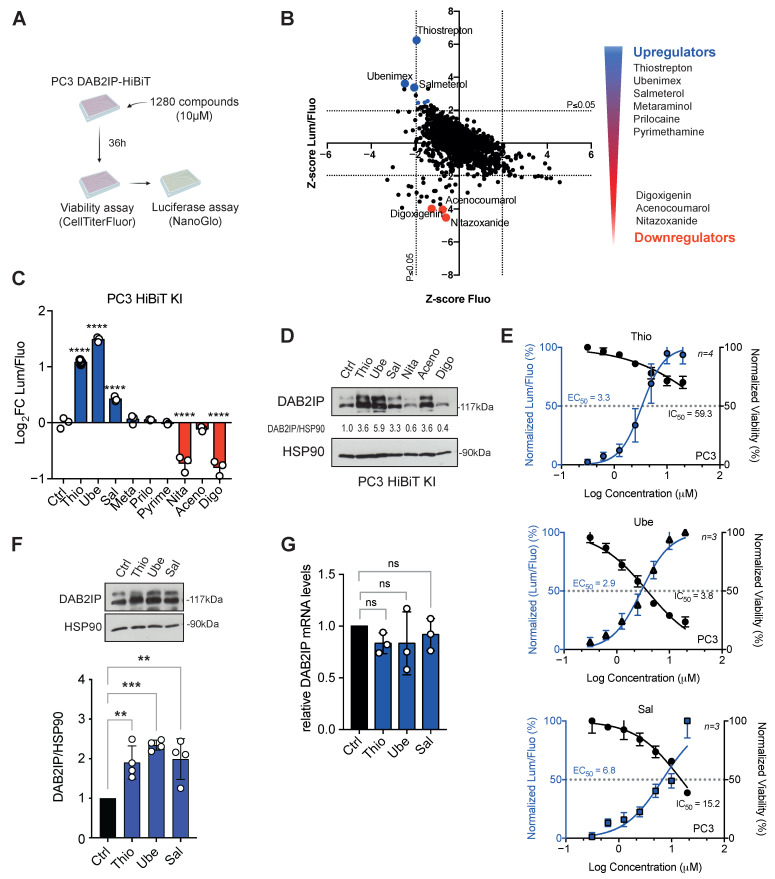
Identification of drugs that modulate DAB2IP protein levels. (**A**) Schematic representation of the high-throughput screening procedure. PC3-HiBiT cells were seeded in 384-well plates, and 24 h later, 1280 compounds were added at 10 μM final concentration. 36 h after treatment, cells were incubated with CellTiterFluor first, and then with NanoGlo lytic reagent. Fluorescence and luminescence readouts were acquired separately (Created with BioRender.com). (**B**) The graph shows the Z-score of luciferase (DAB2IP levels) over fluorescence (cell viability) on the *Y*-axis, plotted against the Z-score of fluorescence (viability) on the *X*-axis. *p*-values of 0.05 were used as thresholds. Hits were selected among compounds falling outside of the *p*-value on the *Y*-axis. On the right, a list of selected drugs modulating DAB2IP levels. (**C**) Histogram summarizes the Lum/Fluo ratio measured with PC3-HiBiT cells seeded in 96-well plates and treated for 36 h with the indicated compounds at 10 μM concentration. Data are mean ± SD of three independent experiments (**** *p* < 0.0001; one-way ANOVA with Dunnett’s post hoc). (**D**) Representative Western blot of endogenous DAB2IP detected in PC3-HiBiT cells treated as in (**C**). HSP90 was blotted as a loading control. Densitometric quantification of the bands is indicated as normalized DAB2IP/HSP90 ratio. (**E**) Dose–response curves of the three best candidate drugs. PC3-HiBiT cells were treated as in (**C**), with variable drug concentrations. The graphs summarize the effect of each drug on DAB2IP levels (Lum/Fluo ratio) and the effect on viability (Fluorescence). The half-maximal effective concentration (EC_50_) and half-maximal inhibitory concentration (IC_50_) are also indicated. Data are mean of *n* independent experiments as indicated in the figure ± SD. (**F**,**G**) Effects of candidate drugs on DAB2IP levels in non-edited parental PC3. (**F**) Cells were treated with the indicated drugs at 10 μM for 36 h. DAB2IP was detected by immunoblotting, with HSP90 as the loading control. Bottom graph: bands were quantified by densitometry, values are mean ± SD of four independent experiments (*** *p* < 0.001, ** *p* < 0.01; one-way ANOVA with Dunnett’s post hoc). (**G**) Cells were treated as in (**F**). DAB2IP mRNA levels were measured by RT-qPCR. Values were normalized on histone H3 and compared to DMSO-treated controls. Data are mean ± SD of three independent experiments (ns = non-significant; one-way ANOVA with Dunnett’s post hoc).

We screened a library containing 1280 compounds, most of which are FDA- or EMA-approved (https://www.prestwickchemical.com). Each compound was added at a 10 μM final concentration to the culture medium in 384-well plates as outlined in Figure 1A. For each drug, the Lum/Fluo ratio was normalized to DMSO-treated controls and expressed as a Z-score. Given the tumor suppressive activity of DAB2IP, we also considered as potential hits compounds that increased the Lum/Fluo ratio with an inhibitory effect on cell viability (Figure 1B). According to these criteria, we selected six positive and three negative regulators that are listed in Figure 1B. In a secondary screen, six molecules were confirmed: thiostrepton, ubenimex, salmeterol as upregulators, and nitazoxamide, acenocoumarol, and digoxigenin as downregulators (Figure 1C).

To corroborate these results, we monitored the effects of the compounds by immunoblotting. As shown in Figure 1D, with five out of six compounds, the variations in DAB2IP protein levels reflected the variations in Lum/Fluo ratio, with the only exception of acenocoumarol, which unexpectedly increased DAB2IP protein levels.

In the context of this study, we focused only on DAB2IP upregulators. As shown in Figure 1E, thiostrepton (Thio) and salmeterol (Sal) displayed similar half-maximal effective concentration (EC_50_) values of 3.3 μM and 2.9 μM, respectively, whereas ubenimex (Ube) had an EC_50_ of 6.8 μM. Concerning the drug effects on viability, only ubenimex markedly affected fluorescence, while thiostrepton and salmeterol displayed half-maximal inhibitory concentrations (IC_50_) significantly above the EC_50_ (Figure 1E).

To rule out clone-specific artifacts, we also tested the compounds on parental non-edited PC3 cells, confirming the results observed in the PC3-HiBiT clone (Figure 1F).

To understand the possible mechanisms underlying drug-induced DAB2IP modulation, we investigated whether their action occurred at the transcriptional level. By RT-qPCR, we observed that all drugs did not significantly change the mRNA levels of DAB2IP, suggesting that they may act on protein synthesis or turnover (Figure 1G).

The top hit compounds were also tested on the other edited prostate cancer cell line, DU145-HiBiT (Figure S3A,B), and on parental non-edited DU145 cells (Figure S3C), confirming their action on DAB2IP. Of note, Thio displayed higher toxicity in DU145 than in PC3; nonetheless, using a lower drug concentration (4 μM), we confirmed that it increased DAB2IP protein levels also in this cell line (Figure S3C). Similarly to what was observed in PC3, even in DU145, the three drugs did not significantly upregulate DAB2IP mRNA (Figure S3D).

Finally, we tested these drugs in a different tumor type. We chose MCF7, a luminal breast cancer cell line, estrogen and progesterone receptor positive, with wild-type p53, since it represents a significantly different model with respect to PC3 and DU145. Also in MCF7, the selected drugs had similar effects on DAB2IP protein levels, without significant upregulation of the transcript (Figure S3E,F). We concluded that Thio, Sal, and Ube can increase endogenous DAB2IP in at least three different cell lines, representing both prostate and breast cancers.

In these experiments, accumulation of DAB2IP was preferentially observed when cells showed minimal or no evidence of drug-induced toxicity, suggesting that this phenomenon is sensitive to cellular stress or damage.

### 3.3. Thio, Sal, and Ube Increase DAB2IP Levels Likely via Different Molecular Mechanisms

The three identified drugs belong to different classes and have different established targets. Thiostrepton is a thiopeptide antimicrobial drug, FDA-approved only for veterinary use, but also known for its anti-tumoral action, mainly ascribable to inhibition of the transcription factor Forkhead box M1 (FOXM1) in mammalian cells [17].

Ubenimex (or Bestatin) is a non-selective inhibitor of aminopeptidases that is clinical trials for the treatment of multiple solid tumors and is currently approved in Japan for the treatment of acute non-lymphocytic leukemia [18,19].

Salmeterol is a highly selective long-acting beta-2 adrenergic agonist (LABA) that is currently prescribed for the treatment of asthma and chronic obstructive pulmonary disease (COPD) [20,21].

**Figure 2:**
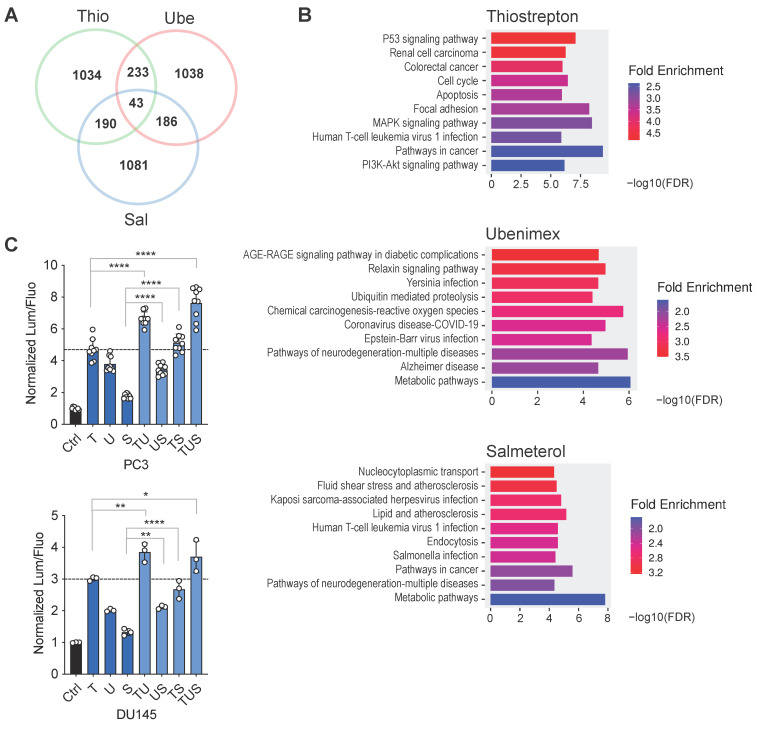
The identified drugs likely act on DAB2IP Via different molecular mechanisms. (**A**) Venn diagram summarizing the top 1500 genes affected by 24 h treatment with 10 μM Thio (T), Ube (U), or Sal (S) in PC3 cells (from ConnectivityMap). (**B**) For each drug, the top 1500 genes were used for gene set enrichment analysis (KEGG pathways) using the ShinyGO platform. (**C**) Thio, Ube, and Sal display an additive effect on DAB2IP upregulation. PC3-HiBiT and DU145-HiBiT cells were treated for 36 h with various combinations of the drugs as indicated, at a total 10 μM final concentration. Endogenous DAB2IP levels were quantified as in Figure 1C. Data are mean ± SD of 3 to 9 wells per condition. Horizontal line indicates the Thio Lum/Fluo reference value. Statistics are shown for selected comparisons (* *p* < 0.05, ** *p* < 0.01, **** *p* < 0.0001; one-way ANOVA with Dunnett’s post hoc).

To investigate whether these drugs may have a common mechanism of action, we used the CLUE platform (https://clue.io) to explore the Broad Institute Connectivity Map. Noticeably, very limited overlaps emerged considering perturbagens and compounds associated with each drug (Figure S4A). We then analyzed the transcriptional effects of these drugs in PC3 and MCF7, the two cell lines in which we obtained functional evidence of DAB2IP upregulation, using the corresponding gene expression data from Connectivity Map. The three drugs have a remarkably different impact on transcription, with Thio modulating a much larger number of genes than Ube and Sal; therefore, for comparative functional annotation, we selected the top 1500 genes differentially affected by each drug. As shown in Figure 2A and Figure S4B, while there was some overlap (14–18%) among the drugs, only a minimal number of DEGs (2.6–2.8%) were shared by all three, both in PC3 (Figure 2A) and MCF7 (Figure S4B). We then used the ShinyGO platform to explore the functional annotation of genes modulated by the drugs. Gene-set enrichment analysis failed to identify obvious common pathways affected by all drugs (PC3 in Figure 2B, MCF7 in Figure S4C), in line with the notion that they have different molecular targets, and probably act on DAB2IP via different mechanisms. Nonetheless, in both cell lines, we detected enrichment for hallmarks such as TNF signaling, hypoxia, KRAS signaling, apoptosis, and EMT (Hallmarks.MsigDB, Figure S5), which are related to known DAB2IP functions [2,3,4].

Finally, using PC3-HiBiT and DU145-HiBiT cell lines, we observed increased accumulation of endogenous DAB2IP when the drugs were used in combination, a result that further supports the idea that these drugs may act on DAB2IP via independent mechanisms (Figure 2C). In the absence of a clear common pathway triggered by the three drugs, in this study, we focused on thiostrepton, given its reported anti-cancer effects [17].

### 3.4. Thiostrepton Does Not Increase DAB2IP Levels by Inhibiting FOXM1

**Figure 3:**
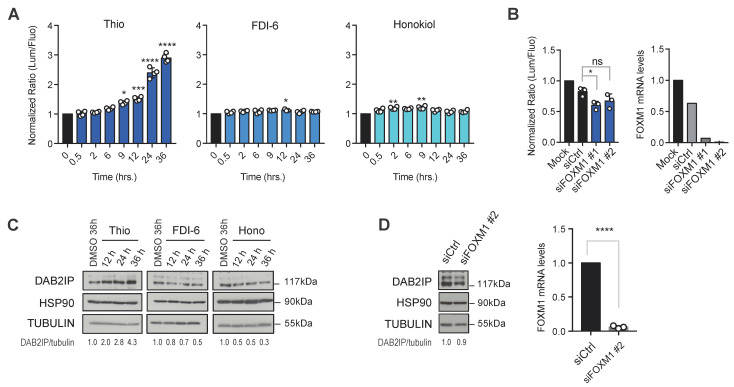
FOXM1 inhibition does not affect DAB2IP levels. (**A**) Two unrelated FOXM1 inhibitors do not increase DAB2IP levels. PC3-HiBiT cells seeded in 96-well plates were treated with 10 μM thiostrepton, FDI-6, or honokiol for the indicated time points, and endogenous DAB2IP levels were measured by Luc/Fluo ratio as in Figure 1C. Data are mean ± SD of three wells per condition (* *p* < 0.05, ** *p* < 0.01, *** *p* < 0.001, **** *p* < 0.0001; one-way ANOVA with Dunnett’s post hoc). (**B**) FOXM1 silencing does not increase DAB2IP levels. Left, PC3-HiBiT cells were transfected for 48 h with two different siRNAs targeting FOXM1, and DAB2IP levels were measured by Luc/Fluo ratio as in A. Right, FOXM1 mRNA levels were measured by RT-qPCR as a control of silencing efficiency. Data are mean ± SD of three wells per condition (ns = non-significant, * *p* < 0.05; one-way ANOVA with Dunnett’s post hoc). (**C**) Parental non-edited PC3 cells were treated as in A. Representative Western blot of endogenous DAB2IP protein, with HSP90 and tubulin as loading controls. Densitometric quantification of the bands is indicated as normalized DAB2IP/tubulin ratio. (**D**) Parental non-edited PC3 cells were transfected for 48 h with FOXM1 siRNA. Left, representative Western blot of endogenous DAB2IP. HSP90 and tubulin were blotted as loading controls. Right, FOXM1 mRNA levels measured by RT-qPCR as a control of silencing. Data are mean ± SD of three independent experiments (**** *p* < 0.0001; unpaired Student’s *t*-test).

One of the most relevant targets of thiostrepton in mammalian cells is the transcription factor FOXM1; Thio has been reported to inhibit its transcriptional activity and its expression levels [22,23]. FOXM1 regulates the expression of genes involved in cellular processes such as cell cycle, DNA repair, senescence, apoptosis, migration, invasion, and drug resistance [24]. We therefore asked whether FOXM1 inhibition would be sufficient to increase DAB2IP protein levels, possibly via an indirect mechanism. For this purpose, we treated PC3-HiBiT cells with two different FOXM1 inhibitors, FDI-6 and the natural compound honokiol [25,26]. Although FDI-6 and honokiol induced a slight increase in Lum/Fluo ratio at certain time points (Figure 3A), neither had an effect comparable to thiostrepton, especially at longer times of treatment (Figure 3A). We also depleted endogenous FOXM1 using two different siRNAs and detected no increase in DAB2IP levels (Figure 3B). We finally confirmed these observations in non-edited PC3, where no increase in DAB2IP protein was observed upon drug treatment (Figure 3C) or siRNA-mediated FOXM1 knockdown (Figure 3D). Together, these results strongly suggest that Thio does not affect DAB2IP levels via inhibition of FOXM1, thus implicating a different molecular mechanism for DAB2IP upregulation, which remains to be uncovered.

### 3.5. The Cancer-Inhibitory Effects of Thiostrepton Are Mediated in Part by DAB2IP Upregulation

**Figure 4:**
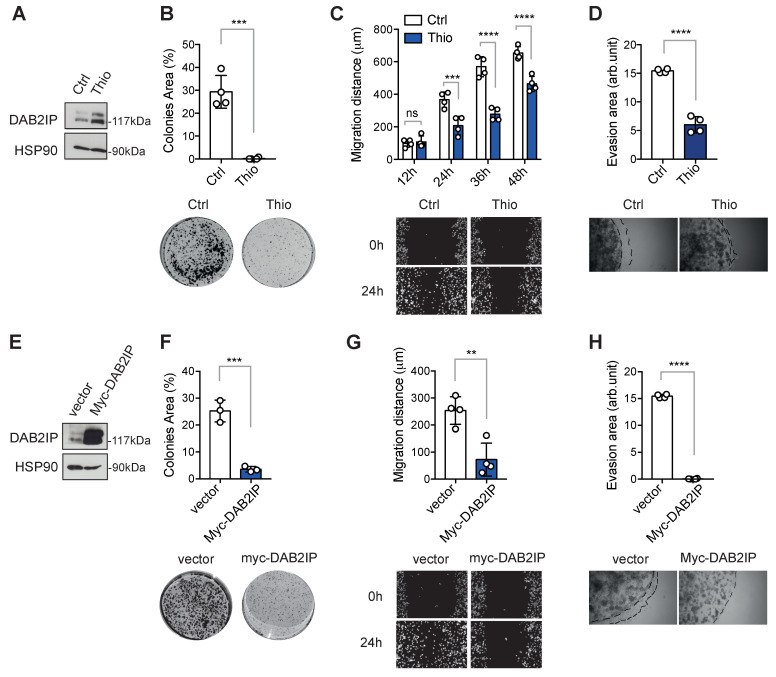
Treatment with thiostrepton inhibits aggressive phenotypes of prostate cancer cells in vitro, an effect similar to DAB2IP overexpression. (**A**) Representative immunoblotting of PC3 cells treated with DMSO (Ctrl) or 3 μM Thio for 48 h, with HSP90 blotted as loading control. (**B**) Thio inhibits colony formation. An identical number of PC3 cells was plated at low density. After 48 h, cells were treated with 3 μM Thio or DMSO (Ctrl) for 48 h; after 10 days, colonies were stained and photographed (representative pictures are shown). Colony formation efficiency was quantified using ImageJ (cells covered area/dish area). Results are mean ± SD of four independent experiments (*** *p* < 0.001; unpaired Student’s *t*-test). (**C**) Thio reduces cell migration. PC3 cells were grown until 90% confluence, then a central region was scraped off with a sterile tip, and cells were treated with 3 μM Thio or DMSO (Ctrl). Wound closure was checked at the indicated time points (representative images are shown). Migration distance was quantified using ImageJ as indicated in Section 2. Results are mean ± SD of four wells per condition (ns = non-significant, *** *p* < 0.001, **** *p* < 0.0001; two-way ANOVA with Sidak correction). (**D**) Thio inhibits cell evasion in Matrigel. Cells were pre-treated for 48 h with 3 μM Thio or DMSO (Ctrl). Then, the same number of cells was seeded inside a Matrigel drop in 0.1% serum, covered with 10% serum medium. Evasion events were monitored during the following days by phase contrast microscopy (representative images at 7 days are shown). The graph summarizes the area of evasion events at 6–7 days quantified using ImageJ and expressed as arbitrary units. Results are mean ± SD of four independent experiments (**** *p* < 0.0001; unpaired Student’s *t*-test). (**E**) Representative immunoblotting of control (empty vector) and DAB2IP-stably expressing PC3 cells, with HSP90 blotted as the loading control. (**F**) DAB2IP overexpression inhibits colony formation. An identical number of PC3 cells stably expressing myc-DAB2IP or empty vector were plated at low density; colony formation efficiency was quantified as in B. Results are mean ± SD of three independent experiments (*** *p* < 0.001; unpaired Student’s *t*-test). (**G**) DAB2IP overexpression reduces cell migration. PC3 cells stably expressing myc-DAB2IP or empty vector were plated at high confluence, and wound-healing assays were performed as in C (representative images are shown). Cell migration was quantified at 24 h, the shortest time point, with a clearly detectable effect of Thio. Results are mean ± SD of four wells per condition (** *p* < 0.01; unpaired Student’s *t*-test). (**H**) DAB2IP inhibits cell evasion in Matrigel. PC3 cells stably expressing myc-DAB2IP or empty vector were seeded in Matrigel drops, and evasion events were monitored and quantified as in (**D**). The results are mean ± SD of four independent experiments (**** *p* < 0.0001; unpaired Student’s *t*-test).

To investigate the impact of Thio on oncogenic phenotypes, we performed proliferation, migration, and invasion assays using PC3 cells. To minimize nonspecific effects due to toxicity, Thio was tested at the EC_50_ concentration. Despite the relatively low concentration, it effectively reduced the proliferative capabilities of prostate cancer cells as assessed by colony formation assay (Figure 4A,B). Moreover, it significantly decreased the migratory and invasive capabilities of PC3, assessed by wound healing and Matrigel invasion assays, respectively (Figure 4C,D).

To compare the effects of Thio with those of DAB2IP overexpression, we stably expressed DAB2IP in PC3 cells. In line with several previous observations, ectopic DAB2IP strongly inhibited colony formation, cell migration, and Matrigel invasion (Figure 4E–H), an effect similar to that observed after Thio treatment.

Next, to assess the potential role of DAB2IP upregulation in the onco-suppressive effects of thiostrepton, we knocked out DAB2IP in PC3 using CRISPR/Cas9 and analyzed the effects of the drug in these cells.

**Figure 5:**
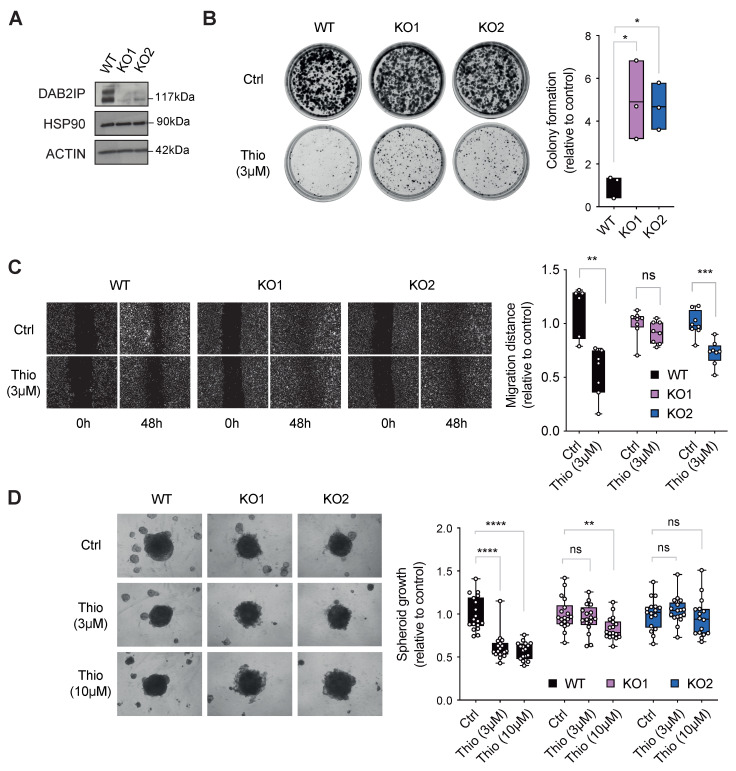
The inhibitory effects of thiostrepton on cancer cells are reduced upon DAB2IP depletion. (**A**) Representative Western blot of DAB2IP expression in parental (WT) and DAB2IP knock-out (KO1 and KO2) PC3 cells, with HSP90 and actin as loading controls. (**B**) Thiostrepton is less effective in suppressing colony formation in the absence of DAB2IP. An identical number of PC3 cells (WT, KO1, and KO2) was plated at low density. After 48 h, cells were treated with 3 μM Thio or DMSO (Ctrl) for 48 h, and then cultured for 10–14 days. Colonies were stained, photographed (representative pictures are shown), and colony formation efficiency was quantified as in Figure 4B. The graph summarizes colony formation in treated samples normalized to their respective controls (DMSO). The results are represented as floating bars from minimum to maximum value, horizontal lines show the mean; *n* = 3 independent experiments (* *p* < 0.05; one-way ANOVA with Dunnett’s post hoc). (**C**) Thiostrepton-mediated inhibition of cell migration is variably reduced in the absence of DAB2IP. PC3 cells (WT, KO1, and KO2) were grown until 90% confluence, then a central region was scraped off with a sterile tip, and cells were treated with 3 μM Thio or DMSO (Ctrl). Wound closure was measured at 48 h (representative images are shown). Migration distance was quantified as in Figure 4C. The results are represented as box plots, horizontal lines show the median, and whiskers extend to the minimum and maximum values. Data represent six to eight wells per condition (ns = non-significant, ** *p* < 0.01, *** *p* < 0.001; Mann–Whitney test). (**D**) Thiostrepton is less effective in suppressing the growth of prostate cancer cell spheroids in the absence of DAB2IP. An identical number of cells (WT, KO1, and KO2) was plated in 96-well ultra-low attachment plates to form spheroids. After 48 h, spheroids were treated with thiostrepton at the indicated concentrations or DMSO (Ctrl). The area of spheroids was measured 6–7 days after treatment (representative images are shown). The results are represented as box plots, horizontal lines show the median, and whiskers extend to the minimum and maximum values; *n* = 3 independent experiments with four to six spheroids per replicate (ns = non-significant, **** *p* < 0.0001, ** *p* < 0.01; two-way ANOVA with Tukey correction).

Strikingly, DAB2IP loss dampened the inhibitory effect of Thio on cell proliferation, as assayed by colony formation (Figure 5A,B). In contrast, DAB2IP loss appeared less involved in the inhibitory effect of Thio on cell migration in wound-healing assays (Figure 5C), at least in one of the knockout cell populations.

To reinforce the above observations, we investigated the effects of Thio on the capability of PC3 to grow into spheroids. Consistently with previous assays, Thio strongly reduced the spheroid growth of parental PC3 (Figure 5D). We next assessed the contribution of DAB2IP to the effect of Thio on spheroid growth. Although knockout cells form spheroids that are morphologically distinct from WT cells—KO spheroids have increased protrusions on the surface—it is evident that thiostrepton had a milder inhibitory effect on 3D growth of DAB2IP-depleted cells (Figure 5D).

Taken together, these results indicate that upregulation of DAB2IP can contribute to the cancer-inhibitory action of thiostrepton, with potential implications for the clinical repurposing of this drug.

## 4. Discussion

The tumor suppressor DAB2IP is frequently downregulated in various human malignancies by multiple mechanisms, but is rarely deleted or mutated. This evidence makes it a good candidate for the development of anti-cancer drugs aimed at increasing its expression. Based on this assumption, we performed a drug-repurposing screen to search for molecules capable of increasing the levels of DAB2IP in cancer cells.

According to our experience, endogenous DAB2IP cannot be reliably detected by immunofluorescence or other imaging techniques suitable for high-throughput screening. To overcome this limitation, we tagged endogenous DAB2IP with the HiBiT peptide for quantitative detection by luminescence. Using a C-terminal insertion site, we tagged all possible isoforms with minimal impact on the structure and function of the protein, so that HiBiT-derived luminescence would theoretically reflect the total abundance of all DAB2IP proteoforms. Such an approach was specifically designed to facilitate the identification of compounds modulating all the steps of endogenous regulation—from transcription to translation, from RNA stability to protein turnover—with high sensitivity.

Our screening identified three molecules able to upregulate endogenous DAB2IP in various cell lines: thiostrepton, ubenimex, and salmeterol. Of note, DAB2IP was found to be upregulated by ubenimex in a high-throughput mass spectrometry survey of drug effects in colon cancer cells (DeepCoverMOA, [27]), an observation that indirectly validates our screening approach.

None of the tested compounds significantly increased DAB2IP mRNA levels, suggesting a post-transcriptional or post-translational mechanism of action. In this regard, DAB2IP has been reported to be degraded by E3 ubiquitin-ligases Skp2 and Smurf1 [28,29] as well as being inhibited by several microRNAs [8,30,31,32]. It is possible that these or other mechanisms are targeted by the identified compounds. Additional studies are needed to further investigate the molecular mechanisms underlying the action of these drugs.

In this study, we focused on thiostrepton, a thiopeptide antibiotic reported to have anti-cancer activity in different models, with the aim of providing corroborative evidence in support of its repositioning for cancer treatment. There is abundant preclinical evidence that thiostrepton can inhibit the proliferation and survival of various cancer cell lines [33,34,35,36], alone or in combination with other drugs (reviewed in [17]). However, the clinical development of thiostrepton is hampered by its poor solubility in water. Specific formulations have been studied to facilitate its systemic delivery and bioavailability; for instance, encapsulation in micelles, liposomes, and other micro- and nano-structures has been shown to improve its delivery and potentiate its anti-cancer properties in mouse models of breast cancer [33]. Also, considerable effort has gone into the development of more soluble thiostrepton derivatives through mutagenesis or chemical synthesis [17]. Notably, a novel formulation of thiostrepton, RSO-021, is currently undergoing a Phase 1/2 clinical trial in patients with malignant pleural effusion from mesothelioma and non-mesothelioma (ClinicalTrials.gov ID: NCT05278975).

Overall, our experiments confirmed that Thio counteracts the aggressiveness of prostate cancer cells in vitro, but also revealed that the Thio effect is less efficient in the absence of DAB2IP, providing the first experimental evidence that DAB2IP upregulation can contribute to the anti-tumor action of this drug.

The anti-cancer properties of thiostrepton are primarily ascribed to the inhibition of the oncogenic transcription factor FOXM1 [23,37,38]. We obtained evidence to reasonably exclude the possibility that DAB2IP upregulation is a result of FOXM1 inhibition, supporting the notion that the two proteins are independent targets of the drug.

The anti-cancer action of Thio is also mediated by the formation of covalent adducts with cysteine residues in the mitochondrial protein peroxiredoxin 3 (PRDX3), an antioxidant enzyme that is frequently upregulated in cancers [39]. Thiostrepton inhibits PRDX3 by crosslinking, causing the accumulation of reactive oxygen species [40,41]. Currently, it is not known whether DAB2IP accumulation is indirectly caused by Thio-induced inhibition of PRDX3 or, as seen with FOXM1, the two proteins are unrelated targets of the drug. Regardless of the mechanism, it is reasonable to speculate that increased DAB2IP levels may support and reinforce the anti-tumor effects of thiostrepton, possibly through synergy with its other molecular targets in a context-dependent manner.

## 5. Conclusions

This study provides evidence that DAB2IP is a potentially druggable molecule and its drug-induced upregulation can counteract various pro-oncogenic features in prostate cancer cell lines. From a pharmacological perspective, our data support previous findings that suggest the repurposing of thiostrepton for cancer treatment, revealing DAB2IP as a possible contributor to its tumor-suppressive activity.

From a methodological perspective, our work confirms and highlights the applicability of the HiBiT system for high-throughput quantification of endogenous proteins that are not easily detected by conventional methods, thereby establishing its effectiveness as a tool for drug discovery applications.

## Figures and Tables

**Table 1:** Sequences of siRNAs/miRNA used.

siRNA/miRNA	Sequence 5′→3′	Purchased From
Control siRNA	Not available	All-Star negative control (1027281, Qiagen, Milano, Italy)
siDAB2IP A	GGAGCGCAACAGUUACCUG	Eurofins MWG
siDAB2IP B	GGUGAAGGACUUCCUGACA	Eurofins MWG
hsa-miR-149-3p	AGGGAGGGACGGGGGCUGUGC	Dharmacon
siFOXM1 #1	GGACCACUUUCCCUACUUU	Integrated DNA Technology (IDT)
siFOXM1 #2	Not available (pool of three siRNAs)	sc-43769, Santa Cruz Biotechnology, Heidelberg, Germany

**Table 2:** Sequences of crRNAs and ssDNA used.

Purpose	crRNA Sequences 5′→3′	PAM
HiBiT KI	CCCTGACCCAGCTGAAAGAG	AGG
DAB2IP KO1	TGTGCTCTATGCCCGCACCA	CGG
DAB2IP KO2	CGGTCACTGTCCACCTGTAC	CGG
**Purpose**	**ssDNA Sequence 5′→3′**	
HiBiT KI	CGTTGGATGCCGCCAATGCCCGCCTCATGAGTGCCCTGACCCAGCTGAAAGGCAGCAGCGGCGTGAGCGGCTGGCGGCTGTTCAAGAAGATTAGCTAGTAGGAGAGGTACAGCATGCAAGCCCGTAACGGCATCTCCCCCACCAACCCCAC	

**Table 3:** List of primary antibodies used.

Antigen	Species	Company	Dilution
DAB2IP	Rabbit (polyclonal)	Bethyl A302-440A, Aurogene, Roma, Italy	1:4000
Myc-tag	Mouse (monoclonal)	9E10 hybridoma supernatant	1:100
HSP90	Mouse (monoclonal)	sc-13119, Santa Cruz Biotechnology, Heidelberg, Germany	1:8000
Actin	Rabbit (polyclonal)	A2066, Merck Life Science, Milano, Italy	1:8000
Tubulin	Mouse (monoclonal)	T5168, Merck Life Science, Milano, Italy	1:8000

**Table 4:** Sequences of PCR primers used.

Target	Sequences 5′→3′
hDAB2IP C-terminal unspliced isoform	Fw: ATCAGCAGGTTGATGTCCGT Rev: TGCAATTTGGTGGGGTTGGT
hDAB2IP C-terminal spliced isoform	Fw: TCAGCAGGTTGATGTCCGTG Rev: TGCGCACGCTCAACTTAAAA
hDAB2IP all isoforms	Fw: CACATCACCAACCACTAC Rev: TCCACCTCTGACATCATC
FOXM1	Fw: ATGCCCAACACGCAAGTAGT Rev: TAGCTGCAGGTTTTGGTCCC
H3	Fw: GTGAAGAAACCTCATCGTTACAGGCCTGGT Rev: CTGCAAAGCACCAATAGCTGCACTCTGGAA
HiBiT KI	Fw: TACCTTCTCTTGCCAGCTGC Rev: GGTAGCTTCCTCCCTCCTCA
DAB2IP KO1	Fw: GGAGCACATCCTGAAGCTGT Rev: CCTTGATGCGGATCATGGGT
DAB2IP KO2	Fw: CCCGTGCACATACAGGACAA Rev: TACCACTTCTCCACGAACTGC

## Data Availability

The original contributions presented in this study are included in the article/Supplementary Materials. Further inquiries can be directed to the corresponding authors.

## Supplementary Materials

The following supporting information can be downloaded at: https://www.mdpi.com/article/10.3390/biom15081147/s1, Figure S1: Characterization of human DAB2IP isoforms and tagging site; Figure S2: Validation of DAB2IP HiBiT-tagged cells for high-throughput screening; Figure S3: Validation of identified drugs in DU145 prostate cancer and MCF7 breast cancer cell lines; Figure S4: The identified drugs likely act on DAB2IP via different molecular mechanisms; Figure S5 Hallmarks enriched in PC3 and MCF7 after drug treatment.

## References

[1] Rawla P. (2019). Epidemiology of prostate cancer. World J. Oncol..

[2] Bellazzo A., Di Minin G., Collavin L. (2017). Block one, unleash a hundred. Mechanisms of DAB2IP inactivation in cancer. Cell Death Differ..

[3] Liu L., Xu C., Hsieh J.-T., Gong J., Xie D. (2016). DAB2IP in cancer. Oncotarget.

[4] De Florian Fania R., Bellazzo A., Collavin L. (2024). An update on the tumor-suppressive functions of the RasGAP protein DAB2IP with focus on therapeutic implications. Cell Death Differ..

[5] Zhang R., He X., Liu W., Lu M., Hsieh J.-T., Min W. (2003). AIP1 mediates TNF-alpha-induced ASK1 activation by facilitating dissociation of ASK1 from its inhibitor 14-3-3. J. Clin. Investig..

[6] Bellazzo A., Collavin L. (2020). Cutting the Brakes on Ras—Cytoplasmic GAPs as Targets of Inactivation in Cancer. Cancers.

[7] Olsen S.N., Wronski A., Castaño Z., Dake B., Malone C., Raedt T.D., Enos M., DeRose Y.S., Zhou W., Guerra S. (2017). Loss of RasGAP Tumor Suppressors Underlies the Aggressive Nature of Luminal B Breast Cancers. Cancer Discov..

[8] Bellazzo A., Di Minin G., Valentino E., Sicari D., Torre D., Marchionni L., Serpi F., Stadler M.B., Taverna D., Zuccolotto G. (2018). Cell-autonomous and cell non-autonomous downregulation of tumor suppressor DAB2IP by microRNA-149-3p promotes aggressiveness of cancer cells. Cell Death Differ..

[9] Di Minin G., Bellazzo A., Dal Ferro M., Chiaruttini G., Nuzzo S., Bicciato S., Piazza S., Rami D., Bulla R., Sommaggio R. (2014). Mutant p53 reprograms TNF signaling in cancer cells through interaction with the tumor suppressor DAB2IP. Mol. Cell.

[10] Valentino E., Bellazzo A., Di Minin G., Sicari D., Apollonio M., Scognamiglio G., Di Bonito M., Botti G., Del Sal G., Collavin L. (2017). Mutant p53 potentiates the oncogenic effects of insulin by inhibiting the tumor suppressor DAB2IP. Proc. Natl. Acad. Sci. USA.

[11] Schwinn M.K., Steffen L.S., Zimmerman K., Wood K.V., Machleidt T. (2020). A Simple and Scalable Strategy for Analysis of Endogenous Protein Dynamics. Sci. Rep..

[12] Lankford K.P., Hulleman J.D. (2024). Protocol for HiBiT tagging endogenous proteins using CRISPR-Cas9 gene editing. STAR Protoc..

[13] Ghetti S., Burigotto M., Mattivi A., Magnani G., Casini A., Bianchi A., Cereseto A., Fava L.L. (2021). CRISPR/Cas9 ribonucleoprotein-mediated knockin generation in hTERT-RPE1 cells. STAR Protoc..

[14] Mittler F., Obeid P., Rulina A.V., Haguet V., Gidrol X., Balakirev M.Y. (2017). High-Content Monitoring of Drug Effects in a 3D Spheroid Model. Front. Oncol..

[15] Min J., Zaslavsky A., Fedele G., McLaughlin S.K., Reczek E.E., Raedt T.D., Guney I., Strochlic D.E., Macconaill L.E., Beroukhim R. (2010). An oncogene-tumor suppressor cascade drives metastatic prostate cancer by coordinately activating Ras and nuclear factor-kappaB. Nat. Med..

[16] Wu K., Xie D., Zou Y., Zhang T., Pong R.-C., Xiao G., Fazli L., Gleave M., He D., Boothman D.A. (2013). The mechanism of DAB2IP in chemoresistance of prostate cancer cells. Clin. Cancer Res..

[17] Bailly C. (2022). The bacterial thiopeptide thiostrepton. An update of its mode of action, pharmacological properties and applications. Eur. J. Pharmacol..

[18] Chen L., Teng Y., Xu W. (2011). Progress in the development of bestatin analogues as aminopeptidases inhibitors. Curr. Med. Chem..

[19] Barnieh F.M., Loadman P.M., Falconer R.A. (2021). Is tumour-expressed aminopeptidase N (APN/CD13) structurally and functionally unique?. Biochim. Biophys. Acta Rev. Cancer.

[20] Cazzola M., Testi R., Matera M.G. (2002). Clinical pharmacokinetics of salmeterol. Clin. Pharmacokinet..

[21] Szczuka A., Wennerberg M., Packeu A., Vauquelin G. (2009). Molecular mechanisms for the persistent bronchodilatory effect of the beta 2-adrenoceptor agonist salmeterol. Br. J. Pharmacol..

[22] Liu S.X., Zhou Y., Zhao L., Zhou L.S., Sun J., Liu G.J., Du Y.S., Zhou Y.N. (2022). Thiostrepton confers protection against reactive oxygen species-related apoptosis by restraining FOXM1-triggerred development of gastric cancer. Free Radic. Biol. Med..

[23] Kwok J.M., Myatt S.S., Marson C.M., Coombes R.C., Constantinidou D., Lam E.W. (2008). Thiostrepton selectively targets breast cancer cells through inhibition of forkhead box M1 expression. Mol. Cancer Ther..

[24] Kalathil D., John S., Nair A.S. (2020). FOXM1 and Cancer: Faulty Cellular Signaling Derails Homeostasis. Front. Oncol..

[25] Halasi M., Hitchinson B., Shah B.N., Váraljai R., Khan I., Benevolenskaya E.V., Gaponenko V., Arbiser J.L., Gartel A.L. (2018). Honokiol is a FOXM1 antagonist. Cell Death Dis..

[26] Gormally M.V., Dexheimer T.S., Marsico G., Sanders D.A., Lowe C., Matak-Vinković D., Michael S., Jadhav A., Rai G., Maloney D.J. (2014). Suppression of the FOXM1 transcriptional programme via novel small molecule inhibition. Nat. Commun..

[27] Mitchell D.C., Kuljanin M., Li J., Van Vranken J.G., Bulloch N., Schweppe D.K., Huttlin E.L., Gygi S.P. (2023). A proteome-wide atlas of drug mechanism of action. Nat. Biotechnol..

[28] Tsai Y.-S., Lai C.-L., Lai C.-H., Chang K.-H., Wu K., Tseng S.-F., Fazli L., Gleave M., Xiao G., Gandee L. (2014). The role of homeostatic regulation between tumor suppressor DAB2IP and oncogenic Skp2 in prostate cancer growth. Oncotarget.

[29] Li X., Dai X., Wan L., Inuzuka H., Sun L., North B.J. (2016). Smurf1 regulation of DAB2IP controls cell proliferation and migration. Oncotarget.

[30] Xu Y., He J., Wang Y., Zhu X., Pan Q., Xie Q., Sun F. (2015). miR-889 promotes proliferation of esophageal squamous cell carcinomas through DAB2IP. FEBS Lett..

[31] Ni Q.F., Zhang Y., Yu J.W., Hua R.H., Wang Q.H., Zhu J.W. (2020). miR-92b promotes gastric cancer growth by activating the DAB2IP-mediated PI3K/AKT signalling pathway. Cell Prolif..

[32] Li X., Zhang X., Zhang Q., Lin R. (2019). miR-182 contributes to cell proliferation, invasion and tumor growth in colorectal cancer by targeting DAB2IP. Int. J. Biochem. Cell Biol..

[33] Wang M., Gartel A.L. (2011). Micelle-encapsulated thiostrepton as an effective nanomedicine for inhibiting tumor growth and for suppressing FOXM1 in human xenografts. Mol. Cancer Ther..

[34] Zhang W., Gong M., Zhang W., Mo J., Zhang S., Zhu Z., Wang X., Zhang B., Qian W., Wu Z. (2022). Thiostrepton induces ferroptosis in pancreatic cancer cells through STAT3/GPX4 signalling. Cell Death Dis..

[35] Ju S.Y., Huang C.Y., Huang W.C., Su Y. (2015). Identification of thiostrepton as a novel therapeutic agent that targets human colon cancer stem cells. Cell Death Dis..

[36] Hansen M.B., Postol M., Tvingsholm S., Nielsen I.O., Dietrich T.N., Puustinen P., Maeda K., Dinant C., Strauss R., Egan D. (2021). Identification of lysosome-targeting drugs with anti-inflammatory activity as potential invasion inhibitors of treatment resistant HER2 positive cancers. Cell. Oncol..

[37] Jiang L., Wu X., Wang P., Wen T., Yu C., Wei L., Chen H. (2015). Targeting FoxM1 by thiostrepton inhibits growth and induces apoptosis of laryngeal squamous cell carcinoma. J. Cancer. Res. Clin. Oncol..

[38] Qiao S., Lamore S.D., Cabello C.M., Lesson J.L., Munoz-Rodriguez J.L., Wondrak G.T. (2012). Thiostrepton is an inducer of oxidative and proteotoxic stress that impairs viability of human melanoma cells but not primary melanocytes. Biochem. Pharmacol..

[39] Ismail T., Kim Y., Lee H., Lee D.-S., Lee H.-S. (2019). Interplay Between Mitochondrial Peroxiredoxins and ROS in Cancer Development and Progression. Int. J. Mol. Sci..

[40] Nelson K.J., Messier T., Milczarek S., Saaman A., Beuschel S., Gandhi U., Heintz N., Smalley T.L., Lowther W.T., Cunniff B. (2021). Unique Cellular and Biochemical Features of Human Mitochondrial Peroxiredoxin 3 Establish the Molecular Basis for Its Specific Reaction with Thiostrepton. Antioxidants.

[41] Cunniff B., Newick K., Nelson K.J., Wozniak A.N., Beuschel S., Leavitt B., Bhave A., Butnor K., Koenig A., Chouchani E.T. (2015). Disabling Mitochondrial Peroxide Metabolism via Combinatorial Targeting of Peroxiredoxin 3 as an Effective Therapeutic Approach for Malignant Mesothelioma. PLoS ONE.

