# Supplementary material for: Drug-Repurposing Screen Identifies Thiostrepton as a Novel Regulator of the Tumor Suppressor DAB2IP
Source: Biomolecules. 2025 Aug 8;15(8):1147. doi: 10.3390/biom15081147 (PMC12384208; doi:10.3390/biom15081147)

Figure 1D

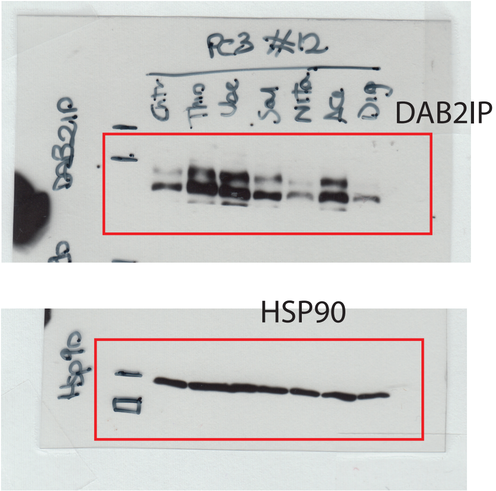


Figure 1F

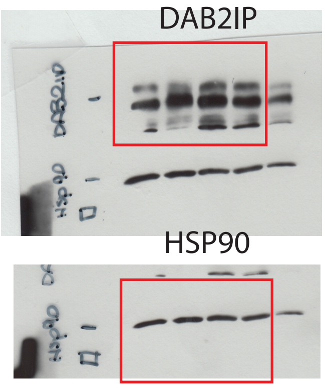

Figure 3C

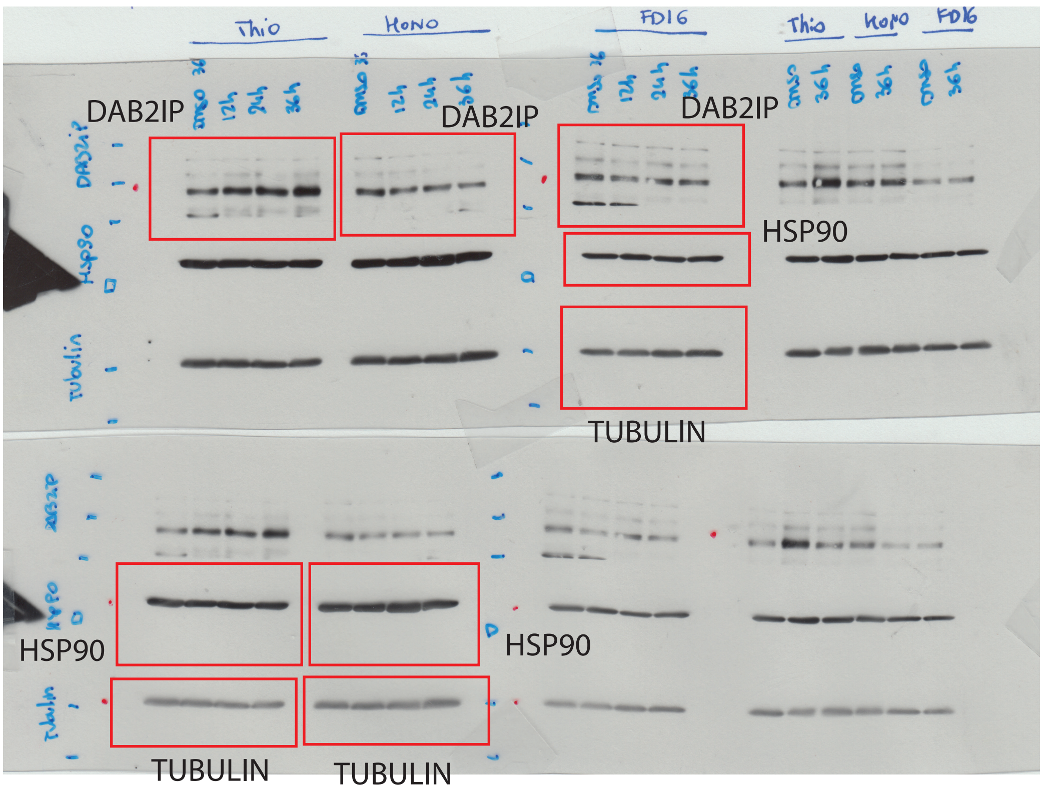


Figure 3D

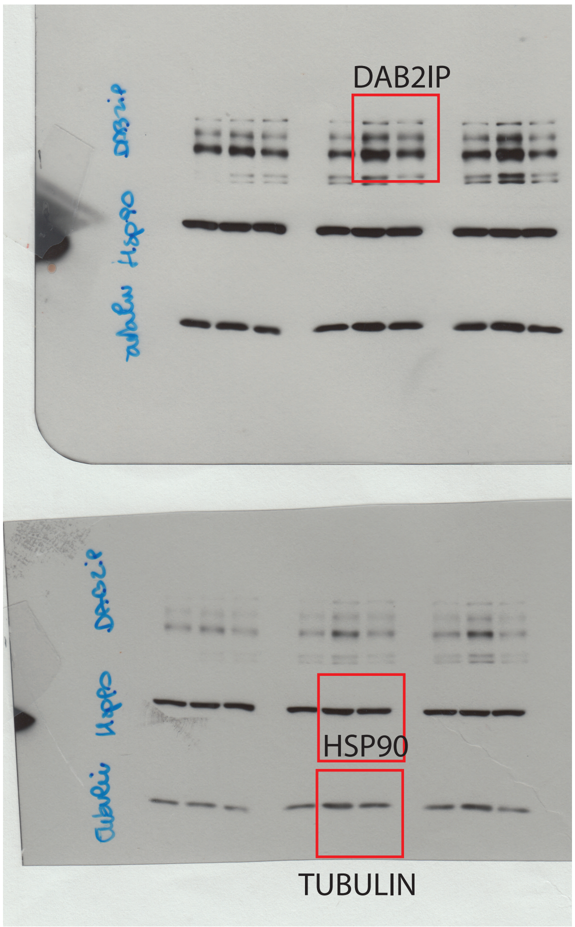

Figure 4A

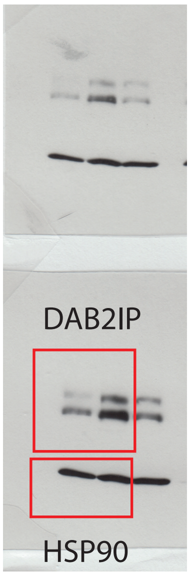


Figure 4E

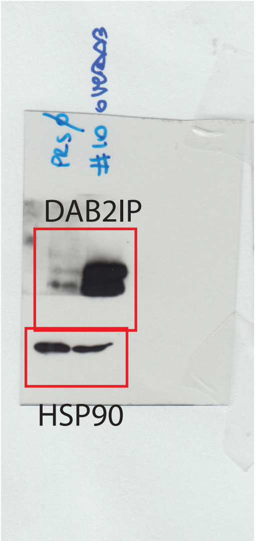


Figure 5A

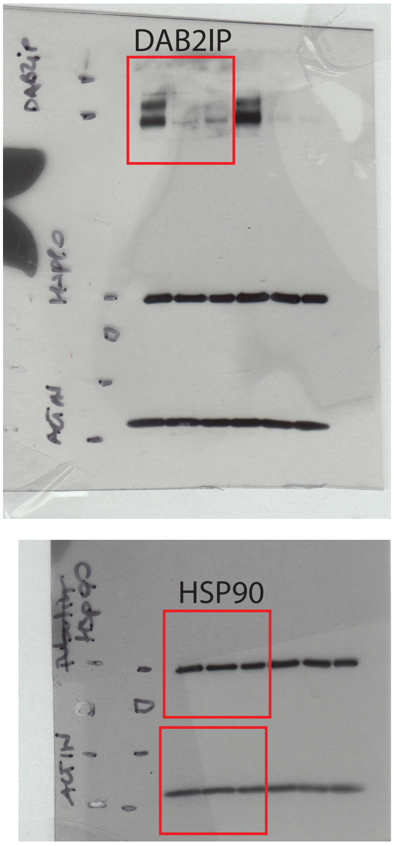


ACTIN

Figure S1D

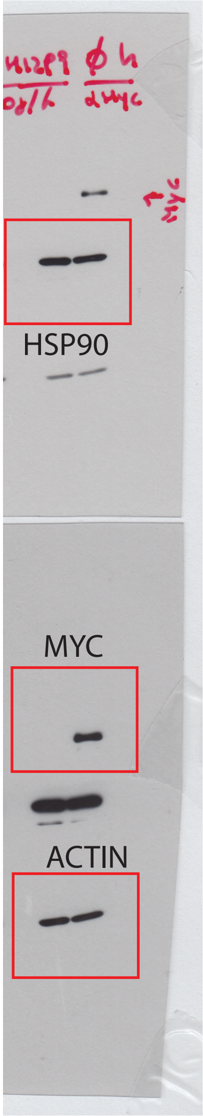


Figure S2D

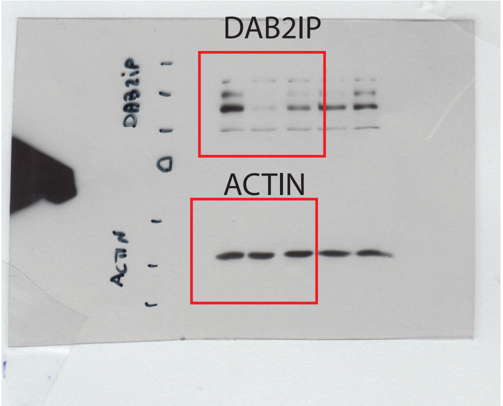


Figure S3C

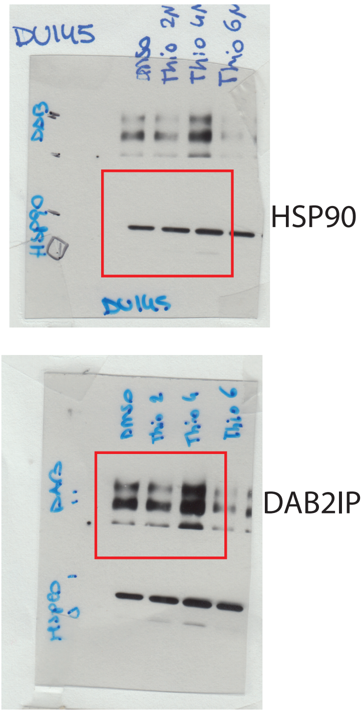


Figure S3C

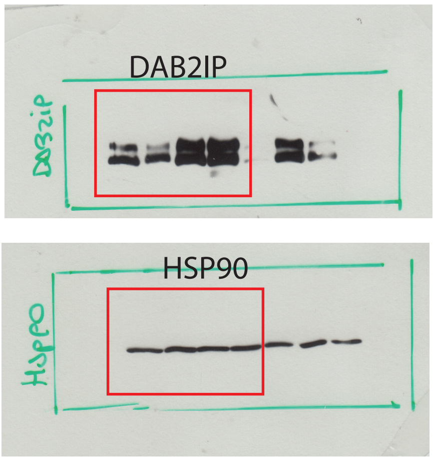


Figure S3E

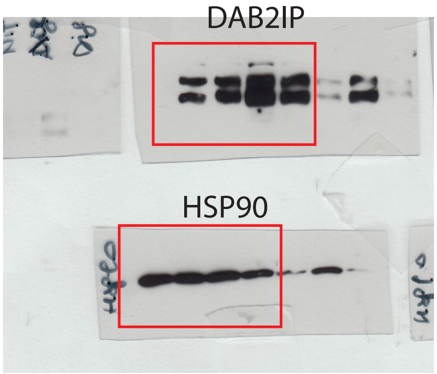

Supplement: Supplementary file 1 [file biomolecules-15-01147-s001.zip › biomolecules-3711020 WB uncropped scans.pdf]
